# Metabolic Reprograming of Cystic Fibrosis Macrophages via the IRE1α Arm of the Unfolded Protein Response Results in Exacerbated Inflammation

**DOI:** 10.3389/fimmu.2019.01789

**Published:** 2019-08-02

**Authors:** Samuel Lara-Reyna, Thomas Scambler, Jonathan Holbrook, Chi Wong, Heledd H. Jarosz-Griffiths, Fabio Martinon, Sinisa Savic, Daniel Peckham, Michael F. McDermott

**Affiliations:** ^1^Leeds Institute of Rheumatic and Musculoskeletal Medicine, University of Leeds, Leeds, United Kingdom; ^2^Leeds Institute of Medical Research at St. James's, University of Leeds, Leeds, United Kingdom; ^3^Leeds Cystic Fibrosis Trust Strategic Research Centre, University of Leeds, Leeds, United Kingdom; ^4^Department of Biochemistry, University of Lausanne, Epalinges, Switzerland; ^5^Department of Clinical Immunology and Allergy, St. James's University Hospital, Leeds, United Kingdom; ^6^Adult Cystic Fibrosis Unit, St. James's University Hospital, Leeds, United Kingdom

**Keywords:** cystic fibrosis, inflammation, metabolism, IRE1, XBP1, UPR, macrophages

## Abstract

Cystic Fibrosis (CF) is a recessive genetic disorder caused by mutations in the cystic fibrosis transmembrane conductance regulator (CFTR). CFTR mutations cause dysregulation of channel function with intracellular accumulation of misfolded proteins and endoplasmic reticulum (ER) stress, with activation of the IRE1α-XBP1 pathway that regulates a subset of unfolded protein response (UPR) genes. This pathway regulates a group of genes that control proinflammatory and metabolic responses in different immune cells; however, the metabolic state of immune cells and the role of this pathway in CF remain elusive. Our results indicate that only innate immune cells from CF patients present increased levels of ER stress, mainly affecting neutrophils, monocytes, and macrophages. An overactive IRE1α-XBP1 pathway reprograms CF M1 macrophages toward an increased metabolic state, with increased glycolytic rates and mitochondrial function, associated with exaggerated production of TNF and IL-6. This hyper-metabolic state, seen in CF macrophages, is reversed by inhibiting the RNase domain of IRE1α, thereby decreasing the increased glycolic rates, mitochondrial function and inflammation. Altogether, our results indicate that innate immune cells from CF patients are primarily affected by ER stress. Moreover, the IRE1α-XBP1 pathway of the UPR is responsible for the hyper-metabolic state seen in CF macrophages, which is associated with the exaggerated inflammatory response. Modulating ER stress, metabolism and inflammation, by targeting IRE1α, may improve the metabolic fitness of macrophages, and other immune cells in CF and other immune-related disorders.

## Introduction

CF is one the most common life limiting autosomal recessive genetic disorder, mainly affecting the lungs and digestive system. CF is caused by mutations in the *CFTR* gene, which regulates ion transport in epithelial, and immune cells. CFTR mutations result in abnormal ion channel function and intracellular accumulation of misfolded proteins leading to ER stress and activation of the IRE1α-XBP1 pathway of the UPR (1). Activation of IRE1α leads to splicing of XBP1 (XBP1s), the latter being a transcription factor involved in the regulation of several cellular pathways, including inflammation, and metabolism (2–4). Inflammation is one of the most common complications of CF, mainly caused by recurrent opportunistic infections, the most common being *Pseudomonas aeruginosa, Staphylococcus aureus*, and *Haemophilus influenza*, leading to the production of several proinflammatory cytokines, such as IL-8, IL-6, IL-1β, IL-18, and TNF (5).

In previous studies, it has been suggested that loss of CFTR function in monocytes and macrophages contributes to the exaggerated inflammatory responses observed in patients with CF (6, 7). Alveolar macrophages are in part responsible for these exaggerated inflammatory responses, partly due to the upregulation of XBP1s inducing high levels of IL-6 production (8). Other studies have reported an increased TLR-4 expression in monocytes from patients with CF, which may also contribute to the hyper-inflammatory state (9, 10). Macrophages are divided broadly into two classes, resident macrophages, and monocyte-derived macrophages, and the latter can be polarized into a proinflammatory (M1) classically activated, or anti-inflammatory (M2) alternatively activated phenotype (11). The degree of M1/M2 macrophage polarization has been reported to be altered in patients with CF, with potential implications for their condition (12, 13). The IRE1α-XBP1 pathway is crucial for M1/M2 macrophage polarization, favoring M1 macrophage polarization while supressing M2 macrophage polarization when overactive (14). Recently, an important role of the IRE1α-XBP1 pathway was described in regulating the metabolic fitness of immune cells (4); however, the metabolic state and the implications of the IRE1α-XBP1 pathway in the pathogenesis of CF have not been fully elucidated. Since atypical activation of the UPR has been reported in CF (15), characterized by high levels of XBP1s and IL-6, we aimed to investigate firstly, whether these UPR abnormalities were present in individual subsets of immune cells, then secondly, the nature of the metabolic conditions of immune cells affected by ER stress and finally, the implications of the IRE1α-XBP1 pathway in the metabolic profile of CF immune cells.

We found that ER stress is present in CF innate immune cells, affecting human bronchial epithelial cells (HBECs), neutrophils, monocytes and M1 macrophages, and is completely absent in the lymphoid cells we studied. We also report a macrophage polarization imbalance, with suppression of M2 macrophage polarization in patients with CF. Even though there were no differences in the proportion of M1 macrophages we found high levels of IL-6 and TNF, with an associated activation of the IRE1α-XBP1 pathway. Previously unreported, we found that the metabolic profile of CF monocytes and M1 macrophages was significantly different to those of healthy controls and these differences could be attributed to activation of the IRE1α-XBP1 pathway. M1 macrophages from CF patients showed elevated glycolic rates and mitochondrial function with an increased glycolytic capacity, glycolytic reserve, mitochondrial respiration, and ATP production. Finally, we showed that the increased metabolic and inflammatory profiles of CF M1 macrophages is primarily due to overactivation of the IRE1α-XBP1 pathway, which could be reversed by inhibition of the RNase domain of IRE1α.

## Materials and Methods

### Contact for Reagent and Resource Sharing

Further information and requests for resources and reagents should be directed to and will be fulfilled by the Lead Contact, Michael F. McDermott (m.mcdermott@leeds.ac.uk).

### Human Subjects

All work involving human samples from patients with CF or HC volunteers was approved by the Health Research Authority, Research Ethics Committees reference 17/YH/0084. Patients with CF were recruited from the Adult Cystic Fibrosis Unit at St. James's University Hospital, Leeds, UK. All patients were diagnosed with CF and had two disease-causing CFTR mutations and clinical features consistent with the diagnosis of CF (Table 1). Patients who were post lung transplant or on CFTR modulators were excluded from the study. Informed written consent was obtained from all participants at the time of the sample collection. Age and sex matched healthy controls were recruited from the St. James's University Hospital, Leeds, UK.

**Table 1:** Patient demographics and clinical data.

	**CF (*n* = 62)**	**HC (*n* = 37)**
Age range (years)	19–50 (32)	21–44 (29)
Male (%)	54.84%	48.65%
**CFTR GENOTYPE**		
DF508/DF508	88.71%	N/A
DF508/621+1 (G>T)	1.61%	N/A
DF508/G551D	1.61%	N/A
W1282X/W1282X	1.61%	N/A
DF508del/c.1521_1523delCTT	4.84%	N/A
3484C>T (p.Arg1162X)/R1162X	1.61%	N/A
BMI (mean)	23.1	N/A
FEV1 (%)	45.9%	N/A

### Cell Lines

The well-characterized human bronchial epithelial cell lines, BEAS2-B (WT), CuFi-1(ΔF508/ΔF508), CuFi-4 (G551D/ΔF508) were obtained from ATCC, all the details regarding these cell lines are deposited in their web page (see table of reagents). All cells were grown on Cell+ surface plates or flasks (Sarstedt). BEAS-2B were cultured in LHC basal medium (ThermoFisher Scientific) supplemented with 10% FBS, 50 U/ml penicillin and 50 μg/ml streptomycin). CuFi-1 and CuFi-4 were cultured in LHC-9 medium (ThermoFisher Scientific), as described by the manufacturer.

### Cell Line Work

Cell lines were seeded at 1 ×10^6^ cells/ml in 6 well plates and cultured in the appropriate growth media. When referred cells were stimulated with LPS (100 ng/ml) and Thapsigargin (Tg) (300 nM) for 4 h. The IRE1α inhibitor, 4 μ8c (50 μM), was used 30 min before stimulation, whenever mentioned. For the detection of IRE1α and pIRE1α, cells were detached, washed, pelleted, fixed, and permeabilized for 20 min, using the Fixation/Permeabilization Kit listed in the table of reagents. Cells were washed twice and stained with IRE1α (Santa Cruz) and pIRE1α (GeneTex) antibodies, or their respective isotype controls, for 30 min. After a final wash the cells were resuspended in brilliant stain buffer (BSB) in FACS collection tubes.

### Human Blood and Isolation of Immune Cells

All blood samples were collected in EDTA pre-coated tubes and processed the same day of collection. PBMCs were isolated from whole blood using a standard density gradient centrifugation method. Blood was mixed with an equal volume of DPBS without Ca^2+^ and Mg^2+^ containing 2% heat inactivated fetal bovine serum (FBS), carefully layered onto of Lymphoprep (Axis-Shield) and centrifuged at 1159xg for 20 min without brakes. The white buffy layer was carefully removed and washed twice in DPBS (2% FBS) by centrifuging at 180xg for 10 min without brakes, to remove platelets. Finally, PBMCs were resuspended in complete RPMI medium (Merck) containing 10% FBS, 50 U/ml penicillin, 50 μg/ml streptomycin and 1% L-Glutamine. 2 ×10^6^ PBMCs from patients with CF or HC volunteers were plated in 6 well plates and stimulated the day after for 4 h with LPS (10 ng/ml). Lymphocytes were isolated from blood using the EasySep Direct Human Total Lymphocyte Isolation Kit (StemCell), plated 2 ×10^6^ cell/ml, and stimulated the day after for 4 h with LPS (10 ng/ml). Neutrophils were isolated from blood using the EasySep Direct Human Neutrophil Isolation Kit (StemCell), plated 3 ×10^6^ cell/ml, and stimulated the same day for 4 h with LPS (10 ng/ml). Monocytes were isolated the same day the PBMCs were obtained using the Pan Monocyte Isolation Kit, human (Miltenyi Biotec), following all the manufacturer instructions, plated 1 ×10^6^ cell/ml and stimulated the day after for 4 h with LPS (10 ng/ml). All cells were cultured in complete RPMI medium (Merck) and kept in a humidified incubator at 37°C, 5% CO_2_.

### M1/M2 Macrophage Polarization

Negatively selected monocytes, as described before, were cultured in complete RPMI medium (Merck) supplemented with either 20 ng/mL human GM-CSF (PeproTech), for M1 differentiation, or 20 ng/mL human M-CSF (PeproTech), for M2 differentiation, and incubated for 6 days adding fresh media with the respective factors on day 3. On day 6 M0 macrophages were activated with 100 ng/mL human IFN-γ (PeproTech) and 50 ng/mL LPS, for M1 macrophage polarization, or 20 ng/mL IL-13 (PeproTech) and 20 ng/mL IL-4 (PeproTech), for M2 macrophage polarization, and incubated for 24 h. M1 macrophages were stimulated with 100 ng/ml LPS for 4 h (Figure 3A). For M1/M2 macrophage characterization using flow cytometry, monocytes seeded at a density of 1 ×10^6^ cell/ml were cultured in tissue culture-treated 6 well plates, stimulated and activated with their respective factors. On day 7 cells were washed twice with DBPS without Ca^2+^ and Mg^2+^, and detached using DPBS with EDTA 10 mM. Cells were washed twice with DPBS (2% FBS), pelleted and resuspended in BSB with human and mouse serum for 30 min on ice. Cells were washed, pelleted, and stained with the surface markers for M1 (CD14+, HLA-DR+, CD274+ and CD86+) and M2-type (CD14+, HLA-DR-, and CD206+) for 30 min on ice. Then, cells were washed, pelleted, fixed, and permeabilized for 20 min on ice. The intracellular markers IL-10 and TNF were then added to fully characterize the M1/M2 macrophages. After a final wash cells were resuspended in BSB in FACS collection tubes. All antibodies used are listed in detail in the table of regents. All the gating strategies are shown in Supplementary Figure 2.

### RNA Analysis

Total RNA isolation was performed using TRIzol reagent and the Phasemaker Tubes (ThermoFisher Scientific) according to the manufacturer's protocol. RNA quality and quantity were further determined by 260/280 and 260/230 ratios using a NanoDrop spectrophotometer. RNA was converted to cDNA using no more than 1 mg of sample with the High-Capacity cDNA Reverse Transcription Kit (ThermoFisher Scientific). XBP1 mRNA splicing was detected by reverse transcription (RT)-PCR using the following set of primers: Forward 5′-CTGAAGAGGAGGCGGAAGC-3′ and reverse 5′-AATACCGCCAGAATCCATGG-3′, which recognize both the XBP1s and XBP1u mRNA. The transcripts were then identified on a 3% agarose gel. Real-time quantitative PCR was done using PowerUp SYBR Green Master Mix reagent or TaqMan universal PCR Master Mix in the QuantStudio 7 Flex Real-Time PCR System (ThermoFisher Scientific) to determine the mRNA levels of the reported genes. mRNA levels were normalized to the levels of HPRT and PPIA RNA transcripts for the cell lines and HPRT for lymphocytes, neutrophils, monocytes and M1 macrophages. All the primers used for qPCR are listed in the Supplementary Table 1. All primers were optimized for specific amplification of the target gene.

### Cytokine Detection

Cytokines levels from cell cultured media were detected by the IL-6, TNF, and IL-10 ELISAs kits listed in the table of reagents, following the manufactures recommendations. In general, ELISA plates were coated with 100 μl cytokine capture antibody in PBS overnight at 4°C. Plates were washed 3 times with PBST (PBS containing 0.5% Tween 20) and the wells blocked in 300 μl assay buffer (0.5% BSA, 0.1% Tween 20 in PBS) by incubating for 1 h. Then, plates were washed twice with PBST and 100 μl of sera/culture supernatants, together with appropriate standards, were added to wells in duplicates. 50 μl of detection antibody was added to all wells, and incubated for 2 h. After incubation, the plates were washed 5 times with PBST and 100 μl of tetramethybenzidine (TMB) substrate solution (Sigma) were added to all wells and incubated for 30 min. The reaction was stopped by adding 100 μl of 1.8N H_2_SO_4_ and absorbance measured at 450 nm and reference at 620 nm. All incubation steps were done at room temperature with continual shaking at 700 rpm.

### Flow Cytometry

The CytoFLEX-S was used for the detection of (PE) IRE1α and (PerCP) pIRE1α in the cell lines. For the characterization of M1/M2 macrophages the surface and intracellular markers, referenced in the M1/M2 macrophage polarization section, were used and analyzed in the BD Bioscience FACS Calibur. Compensation was done by fluorescence minus one (FMO) for all the antibodies. The pIRE1α (GeneTex) and its Rabbit IgG isotype control (GeneTex) antibodies were conjugated using the LYNX Rapid PerCP Antibody Conjugation Kit, as per the manufacture's recommendations.

### Protein Detection of XBP1s

For the detection of XBP1s and HPRT protein, samples were prepared in RIPA lysis buffer (10 mM Tris-Cl, 1 mM EDTA, 1% Triton X-100, 0.1% sodium deoxycholate, 0.1% SDS and 140 mM NaCl) with phosphatase and protease inhibitors listed in the table of reagents and heated at 95°C for 5 min. Protein concentration was determined using the Pierce BCA Protein Assay Kit. Equal protein concentration was loaded and resolved by 10% SDS-PAGE on Tris-glycine gels and then transferred to Hybond PVDF membranes. Following electrotransfer in transfer buffer (25 mM Tris, 192 mM glycine, pH 8.3, 20% methanol) at 100 V for 1 h, the membranes were blocked for 1 h in blocking solution (PBS containing 0.1% Tween 20 and 5% (w/v) non-fat milk). After 3 washes in PBST (PBS with 0.5% Tween 20), primary antibodies were incubated with PVDF membrane overnight at 4°C. The membrane was washed 3 times with PBST and secondary antibody HRP-linked antibody was added and incubated for 1 h with constant rocking at room temperature. The membrane was washed 5x with PBST and 3 ml of ECL detection system (Merck) were added to the membrane for 1 min, before being imaged with the ChemiDoc Imaging system (Bio-Rad). Primary antibodies used, purified anti-XBP-1s Antibody (BioLegend) at 1/250 dilution, HPRT Antibody (Santa Cruz) at 1/500 dilution. Secondary antibody used, Goat anti-Rabbit IgG (H+L) Poly-HRP Secondary Antibody (ThermoFisher Scientific), was diluted at 1/5000 in PBST.

### Metabolic Experiments for ECAR and OCR

Negatively isolated monocytes, as previously described, were seeded at a density of 1.0 × 10^5^ in XF96 cell-culture microplates previously coated with CellTak (Corning), according to the manufacturer's instructions, and wait 1 h for cellular adherence. Immediately after, ECAR and OCR measurements were analyzed on an XFe96 Extracellular Flux Analyzer (Agilent). For the experiments involving M1 macrophages, initially cells were grown as described in the M1/M2 macrophage polarization section; on day 6, cells were detached using Accutase (ThermoFisher Scientific) and seeded at a density of 3.0 × 10^4^ in XF96 cell-culture microplates. The inhibitory effects of 4 μ8c 50 μM (Merck) and MKC-3,946 10 μM (Cayman Chemical) were examined by pre-treating macrophages 30 min before the addition of LPS and IFNγ, then the cells were cultured for a further 24 h in complete RMPI medium for full activation. After activation, the supernatants were collected for cytokine detection, cells were washed twice and media was changed for Agilent seahorse XF base medium containing 10 mM Glucose (only for the Mito Stress Kit), 2 mM L-glutamine, 1 mM sodium pyruvate, and 1 mM HPES. ECAR and OCR measurements were analyzed on an XFe96 Extracellular Flux Analyzer (Agilent). Once basal ECAR and OCR measurements were obtained, ECAR changes were recorded in response to glucose (10 mM), oligomycin (1 μM), 2-Deoxy-D-glucose (2-DG, 50 mM) following the instructions stated in the XF Cell Glycolysis Stress Test Kit (Agilent); or stimulated with oligomycin (1 μM), FCCP (1 μM), and rotenone/antimycin A (0.5 μM) following the instructions stated in the XF Cell Mito Stress Test Kit (Agilent). All the metabolic parameters were calculated as follow. For the XF Cell Glycolysis Stress test: glycolysis = (maximum rate measurement after glucose stimulation)–(rate measurement before glucose stimulation); glycolytic capacity = (maximum rate measurement after glucose stimulation)–(maximum rate measurement after oligomycin stimulation); glycolytic reserve = (maximum rate measurement after oligomycin stimulation)–(rate measurement before oligomycin stimulation). For the XF Cell Mito Stress test: basal respiration = (measurement before oligomycin stimulation)–(rate measurement after rotenone/antimycin A stimulation); proton leak = (minimum rate measurement after oligomycin stimulation)–(minimum measurement after rotenone/antimycin A stimulation); maximal respiration = (maximum rate measurement after FCCP stimulation)–(minimum rate measurement after rotenone/antimycin A stimulation); reserve respiratory capacity = (maximal respiration)–(basal respiration); ATP production = (basal respiration)–(minimum rate measurement after oligomycin stimulation). Normally 3–5 technical replicates per sample were examined. Immediately after the metabolic analysis, cells were fixed for 10 min in methanol/acetone (4:1) and cell number of each well was determined by nuclear DNA staining with DAPI (BD Biosciences), ECAR and OCR values were normalized accordingly.

### Quantification and Statistical Analysis

All statistical details of experiments are described in each of the figure legends. GraphPad Prism 7 software was used to do all analyses. Data are presented as mean ± SEM. Statistical significance determined using the Bonferroni-Dunn correction method for unpaired independent student's *t*-test. *P*-values of <0.05 were considered to be statistically significant. Nonparametric tests were used to compare the medians between groups.

**Table:** Table of Reagents

**Reagents**	**Source**	**Identifier**
**ANTIBODIES**		
IRE1 alpha (phospho Ser724)	GeneTex	Cat# GTX63722; RRID: N/A
Rabbit IgG isotype control	GeneTex	Cat# GTX35035; RRID: N/A
IRE1α Antibody (B-12) PE	Santa Cruz	Cat# sc-390960-PE; RRID: N/A
Normal mouse IgG1 PE (Isotype)	Santa Cruz	Cat# sc-2866; RRID: AB_737219
V500 Mouse Anti-Human CD14	BD Biosciences	Cat# 561391; RRID: AB_10611856
Anti-HLA-DR PerCP	BD Biosciences	Cat# 347402; RRID: N/A
FITC Mouse Anti-Human CD206	BD Biosciences	Cat# 551135; RRID: AB_394065
PE Rat Anti-Human IL-10	BD Biosciences	Cat#; RRID: AB_397227
APC Mouse Anti-Human CD274	BD Biosciences	Cat# 563741; RRID: AB_2738399
PE-Cy7 Mouse Anti-Human CD86	BD Biosciences	Cat# 561128; RRID: AB_10563077
BV421 Mouse Anti-Human TNF	BD Biosciences	Cat# 562783; RRID: AB_2737790
Purified anti-XBP-1s Antibody	BioLegend	Cat# 619501; RRID: AB_315907
HPRT Antibody (FL-218)	Santa Cruz	Cat# sc-20975; RRID: N/A
Goat anti-Rabbit IgG (H+L) Poly-HRP Secondary Antibody	ThermoFisher Scientific	Cat# 32260; RRID: AB_1965959
**BIOLOGICAL SAMPLES**		
Human Blood Samples	St. James's University Hospital	Health Research Authority REC reference 17/YH/0084
Chemicals, Peptides, and Recombinant Proteins
Lymphoprep	Axis Shield	Cat# 1114544
EasySep Direct Human Total Lymphocyte Isolation Kit	StemCell	Cat# 19655
EasySep Direct Human Neutrophil Isolation Kit	StemCell	Cat# 19666
Pan Monocyte Isolation Kit, human	Miltenyi Biotec	Cat# 130-096-537
Recombinant Human GM-CSF	PeproTech	Cat# 300-03
Recombinant Human M-CSF	PeproTech	Cat# 300-25
Recombinant Human IFN-γ	PeproTech	Cat# 300-02
Recombinant Human IL-13	PeproTech	Cat# 200-13
Recombinant Human IL-4	PeproTech	Cat# 200-04
LPS	InvivoGen	Cat# tlrl-3pelps
4μ8c	Merck	Cat# 412512
MKC-3946	Cayman Chemical	Cat# 19152
PowerUp SYBR Green Master Mix	ThermoFisher Scientific	Cat# A25780
TaqMan Universal PCR Master Mix	ThermoFisher Scientific	Cat# 4304437
TRIzol Reagent and Phasemaker Tubes Complete System	ThermoFisher Scientific	Cat# A33251
Thapsigargin	Merck	Cat# T9033
Tunicamycin	Cell Signaling Technologies	Cat# 12819S
PhosSTOP	Merck	Cat# 4906845001
Pierce Protease Inhibitor Mini Tablets	ThermoFisher Scientific	Cat# A32955
Immobilon Western Chemiluminescent HRP Substrate	Merck	Cat# WBKLS0500
**COMMERCIAL ASSAYS**		
High-Capacity cDNA Reverse Transcription Kit	ThermoFisher Scientific	Cat# 4368814
ELISA IL-6 Human	ThermoFisher Scientific	Cat# CHC1263
ELISA TNF alpha Human	ThermoFisher Scientific	Cat# CHC1753
ELISA IL-10 Human	ThermoFisher Scientific	Cat# CHC1323
LYNX Rapid PerCP Antibody Conjugation Kit	Bio-Rad	Cat# LNK072PERCP
Glycolysis Stress Test Kit	Agilent Technologies	Cat# 103020-100
Pierce BCA Protein Assay Kit	ThermoFisher Scientific	Cat# 23225
**CELL LINES**		
BEAS-2B cell line	ATCC	ATCC CRL-9609
CuFi-1 cell line	ATCC	ATCC CRL-4013
CuFi-4 cell line	ATCC	ATCC CRL-4015
**SOFTWARE**		
GraphPad Prism7	Graphpad software	N/A
CytExpert Software	Beckman Coulter	N/A
Flow Jo Vx0.7	FlowJo, LLC	N/A
Agilent Seahorse Wave	Agilent Technologies	N/A

## Results

### ER Stress and UPR Activation in Different Immune Cells

**Figure 1:**
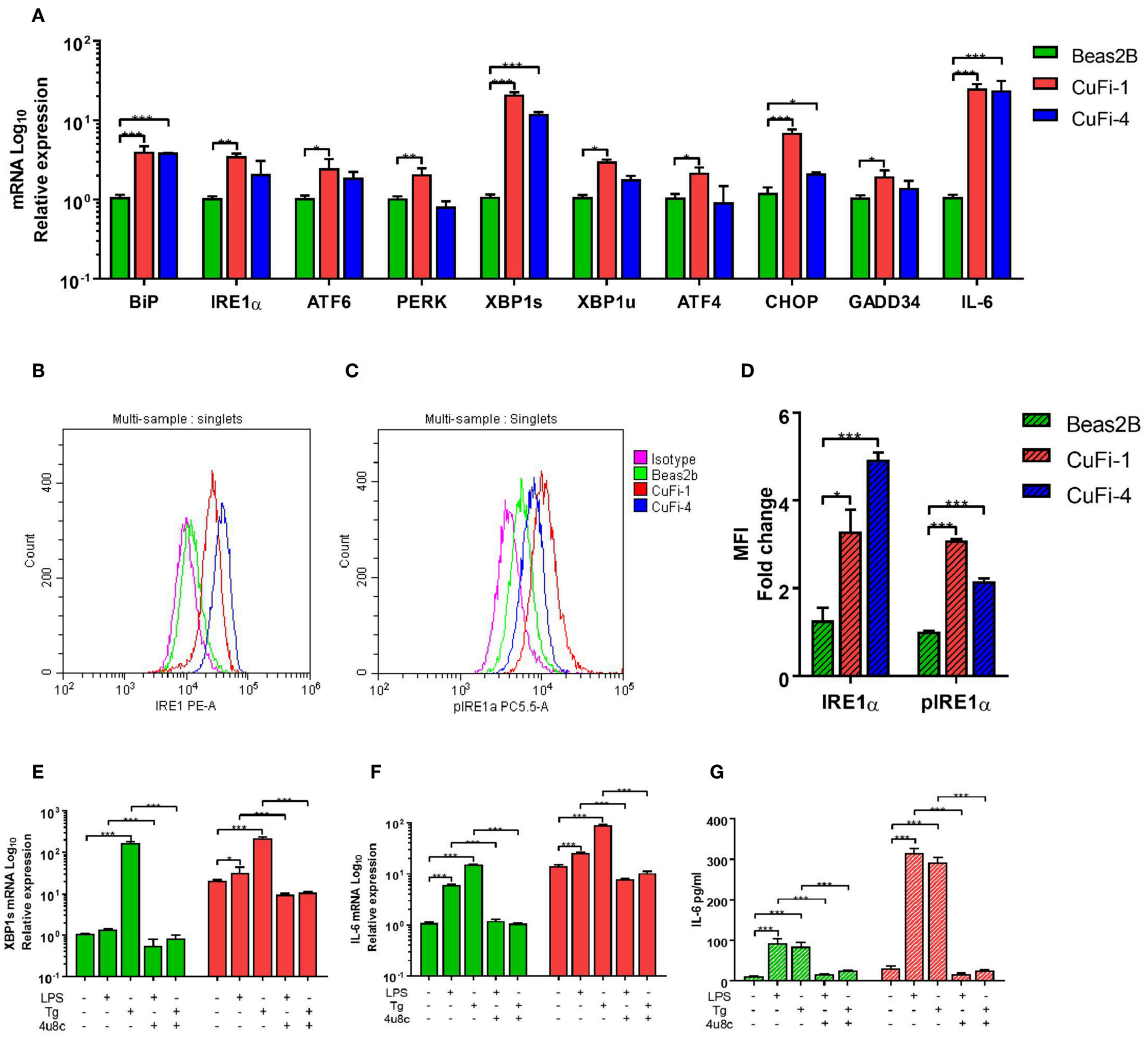
ER Stress and UPR activation in CF HBECs. **(A)** mRNA relative expression of ER stress and UPR markers BiP, IRE1α, ATF6, PERK, XBP1s, XBP1u, ATF4, CHOP, GADD34, and IL-6 in BEAS-2B, CuFi-1, and CuFi-4 cell lines. **(B–D)** Single cells were gated and used to measure the mean fluorescent intensity of each cell line though flow cytometry; IRE1α **(B)** and phosphorylated IRE1α **(C)** protein expression was measured with monoclonal conjugated antibodies (PE, PerCP) in BEAS-2B, CuFi-1, and CuFi-4 cell lines **(D)**. All antibodies were normalized with their respective isotype controls, in each cell line. **(E–G)** XBP1s and IL-6 were measured in response to LPS (100 ng/ml) and Tg (300 nM) for 4 h measuring XBP1s mRNA (E), IL-6 mRNA **(F)** and IL-6 cytokine levels **(G)**; when referred, the IRE1α inhibitor, 4 μ8c (50 μM), was used 30 min before each stimulation. All data is presented as mean ± SEM and mRNA data represented by logarithmic scale base 10. Statistical significance was determined using 2way-ANOVA, Dunnett's test **(A)**, unpaired **(D)** or paired **(E–G)** independent student's *t*-test. ^*^p < 0.05, ^**^p < 0.01, ^***^p < 0.001. *n* = 4 biological replicates for all cell lines.

We primarily analyzed ER stress and UPR activation in Human Bronchial Epithelial Cells (HBECs), as these cells are the first line of defense in the lung and important players in the pathogenies of CF. Three main UPR independent pathways have been described, with the already mentioned IRE1α-XBP1 pathway, the PERK arm responsible for the activation of ATF4, GADD34, and CHOP, and the less well characterized ATF6 (16). We found that CF HBECs harboring class II (ΔF508/ΔF508) and class III (G551D/ΔF508) mutations showed an increase in ER stress, mainly by upregulation of *BiP, XBP1s, CHOP* and *IL-6* transcripts, as described before (Figure 1A) (15). Interestingly, CF HBECs bearing two copies of the ΔF508 mutation not only showed upregulation of the transcripts aforementioned, but also *IRE1*α*, ATF6, PERK, XBP1u, ATF4*, and *GADD34* (Figure 1A). We also found significantly higher levels of IRE1α, at the protein level, and increased phosphorylation of IRE1α (pIRE1α) in the CF HBECs compared to healthy HBECs (Figures 1B–D). As previously mentioned, XBP1s activates the transcription of proinflammatory cytokines, such as IL-6 and TNF. In line with other publications, we confirmed that LPS-induced XBP1s drives the production of IL-6, which can be abrogated by the IRE1α inhibitor, 4 μ8c, in CF HBECs (Figures 1E–G) (8).

**Figure 2:**
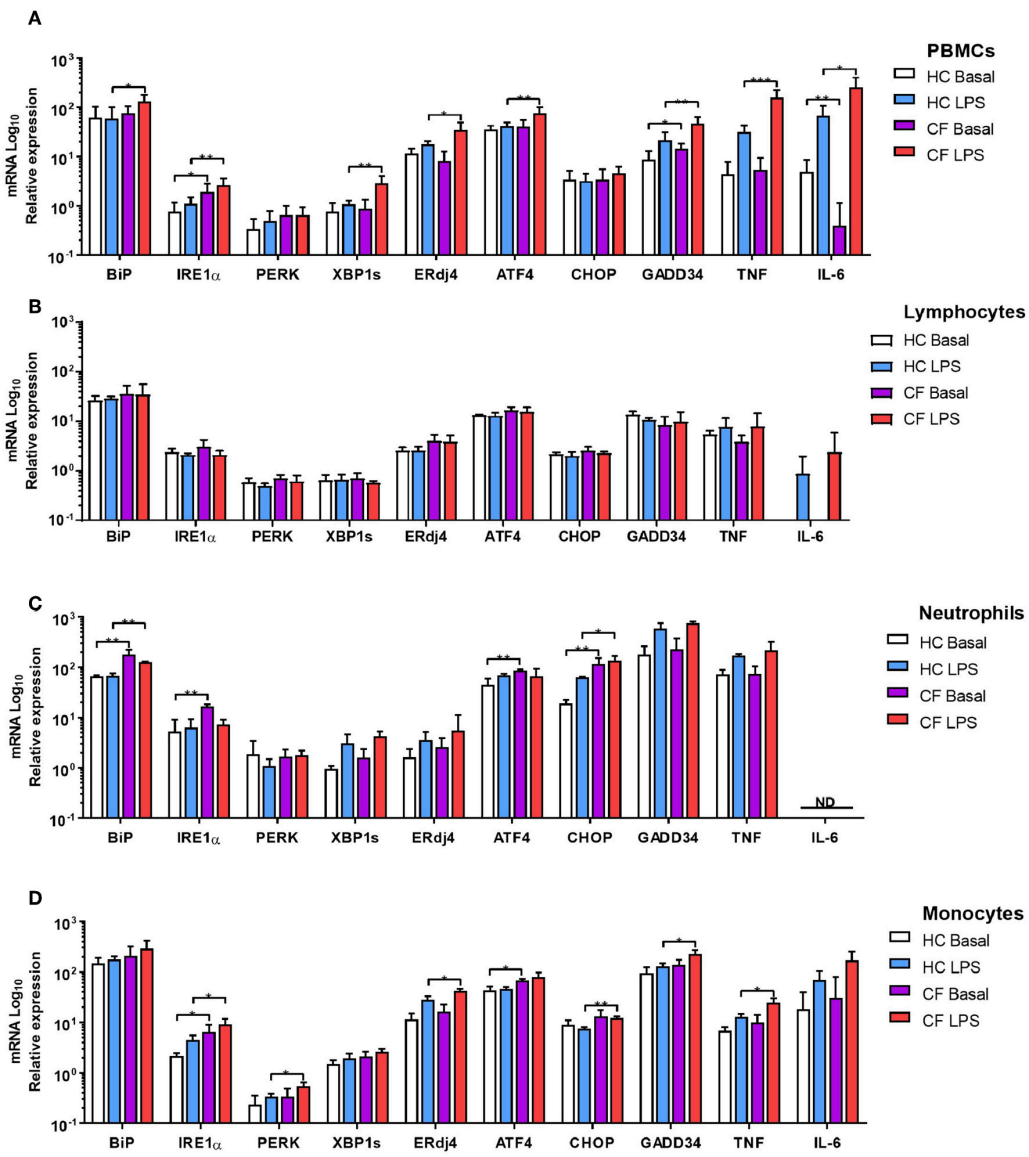
ER stress and UPR activation in immune cells. mRNA relative expression of ER stress and UPR markers *BiP, IRE1*α*, PERK, XBP1s, ERdj4, ATF4, CHOP, GADD34, TNF*, and *IL-6*. **(A)** Primary PBMCs from HC volunteers (*n* = 8) and patients with CF (*n* = 14) at basal conditions and stimulated with LPS (10 ng/ml) for 4 h. **(B)** Isolated lymphocytes from HC volunteers (*n* = 6) and patients with CF (*n* = 6) at basal conditions and stimulated with LPS (10 ng/ml) for 4 h. **(C)** Freshly isolated Neutrophils from HC volunteers (*n* = 6) and patients with CF (*n* = 6) at basal conditions and stimulated with LPS (10 ng/ml) for 4 h. **(D)** Isolated monocytes from HC volunteers (*n* = 6) and patients with CF (*n* = 6) at basal conditions and stimulated with LPS (10 ng/ml) for 4 h. The 14 CF patients and 8 HC volunteers in **(A)** are independent from the other panels. The 6 CF patients and 6 HC volunteers in **(B–D)**, are the same. All data are represented by logarithmic scale base 10 and presented as mean ± SEM. All n values represent biological independent samples. Statistical comparisons were performed by unpaired independent student's *t*-test, ^*^p < 0.05, ^**^p < 0.01, ^***^p < 0.001; ND, Not detected.

There is evidence of an atypical activation of the UPR in Peripheral Blood Mononuclear Cells (PBMCs) from patients with CF under basal conditions (15); however, there is no data showing how PBMCs respond upon bacterial challenges or any other type of ER stressor. Therefore, we analyzed ER stress and UPR activation in PBMCs from patients with CF, at basal conditions, after LPS challenge, and under ER stress conditions induced by Tunicamycin (Tn) and Thapsigargin (Tg). At basal conditions *IRE1*α and *GADD34* were significantly upregulated in CF PBMCs, and *IL-6* downregulated (Figure 2A); however, after stimulation with LPS we detected significant upregulation in *BiP, IRE1*α*, XBP1s, ERdj4, ATF4, GADD34, TNF*, and *IL-6*, compared to healthy controls (HC) volunteers (Figure 2A). Furthermore, we detected similar results after ER stress induction with Tn by a significant upregulation of *BiP* and *IRE1*α, and after stimulation with Tg with a significant higher expression in *PERK* and *XBP1s* (Supplementary Figures 1A–H). We then investigated whether these UPR abnormalities were still present in individual subsets of immune cells, as PBMCs are a mixed population of lymphoid and myeloid cells. No differences were detected in lymphoid cells from patients with CF compared to HC (Figure 2B); however, CF neutrophils and monocytes presented upregulation of several ER stress markers (Figures 2C,D). CF neutrophils presented higher levels of *BiP, IRE1*α*, ATF4*, and *CHOP* at basal conditions, whereas only *BiP* and *CHOP* were significantly upregulated after LPS stimulation (Figure 2C). CF monocytes showed higher levels of *IRE1*α and *ATF4* at basal conditions, and *IRE1*α*, PERK, ERdj4, CHOP, GADD34*, and *TNF* after LPS stimulation (Figure 2D). These results indicate that ER stress and UPR activation is only present in CF innate immune cells and, moreover, it can be influenced by LPS stimulation. Furthermore, this UPR activation is differentially activated in HBECs, neutrophils and monocytes, with a consistent upregulation of *IRE1*α.

### Polarization of M1/M2 Macrophages

**Figure 3:**
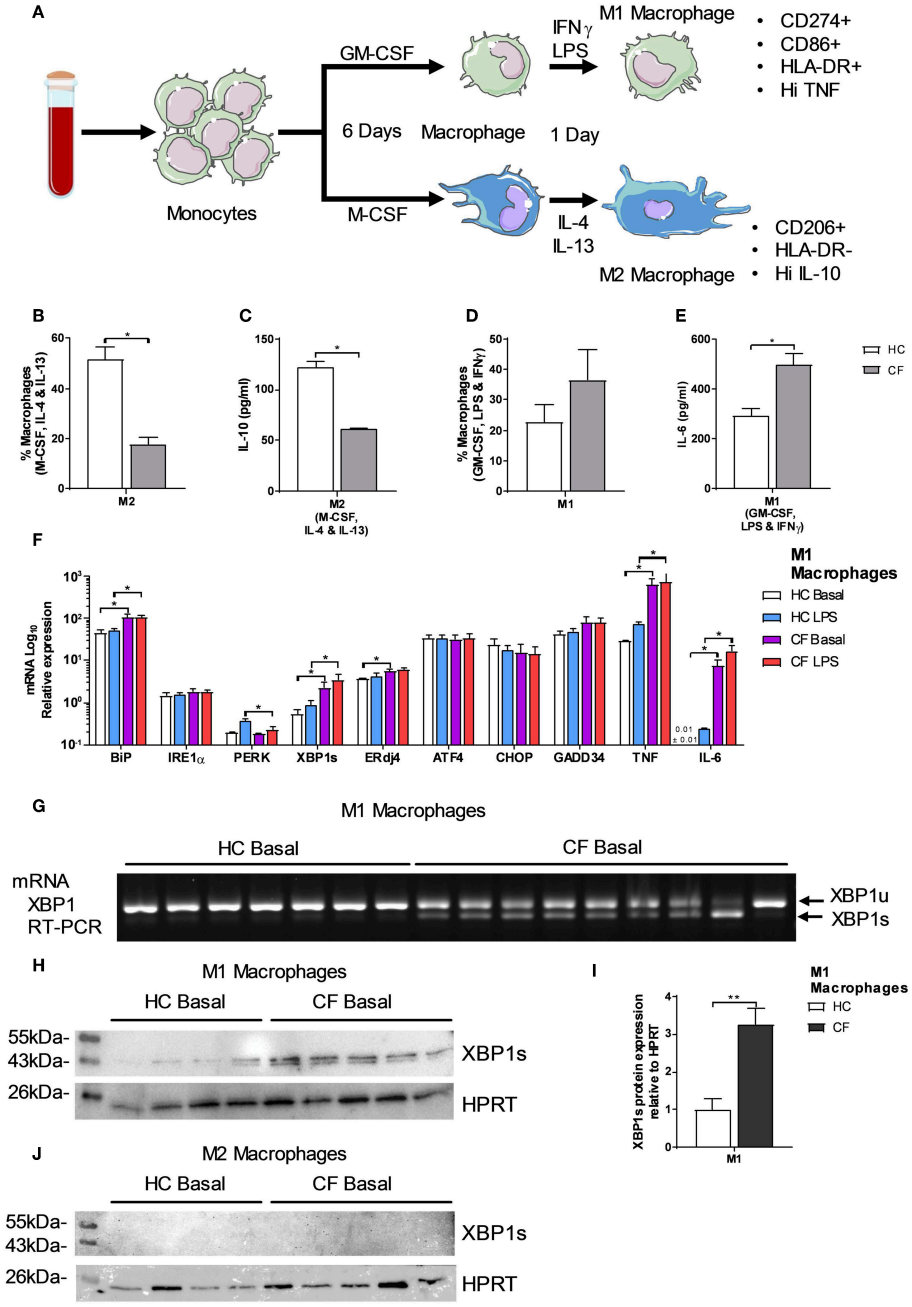
Polarization of M1/M2 Macrophages and XBP1s expression. **(A)** Monocytes were differentiated into macrophages for 6 days with GM-CSF 20 ng/ml or M-CSF 20 ng/ml, then M1-type macrophage activation was achieved by supplementing growth media with 100 ng/ml human IFNγ, and 50 ng/ml LPS. M2-type macrophage activation was achieved by supplementing growth media with 20 ng/ml IL-13 and 20 ng/ml IL-4. Macrophages were characterized as M1-type (markers- CD14^+^, HLA-DR^+^, CD274^+^, CD86^+^ TNF^HI^) or M2-type (markers- CD14^+^, HLA-DR^−^, CD206^+^ and IL-10^HI^). **(B)** Polarized M2 macrophages (M-CSF, IL-4, and IL-13) from HC volunteers (*n* = 7) and patients with CF (*n* = 7) are presented as percentage M1 or M2 of total macrophages measured by flow cytometry. **(C)** IL-10 cytokine levels from M1 and M2 polarized macrophages measured by ELISA. **(D)** Polarized M1 macrophages (GM-CSF, LPS, and IFNγ) from HC volunteers (*n* = 7) and patients with CF (*n* = 7) are presented as percentage M1 or M2 of total macrophages measured by flow cytometry. **(E)** IL-6 cytokine levels from M1 and M2 polarized macrophages measured by ELISA. **(F)** mRNA relative expression of ER stress and UPR markers *BiP, IRE1*α*, PERK, XBP1s, ERdj4, ATF4, CHOP, GADD34, TNF*, and *IL-6* in M1 macrophages from HC volunteers (*n* = 9) and patients with CF (*n* = 9) at basal conditions and stimulated with LPS (100 ngml) for 4 h. **(G)** XBP1s by RT-PCR in M1 macrophages from HC volunteers (*n* = 7) and CF patients (*n* = 9). **(H,I)** XBP1s levels in HC volunteers (*n* = 4) and CF (*n* = 5) M1 macrophages, cells were lysed and immunoblotted for XBP1s and HPRT **(H)**, XBP1s was normalized to HPRT and the ratios were compared to the levels of HC **(I)**. **(J)** XBP1s levels in HC volunteers (*n* = 4) and CF (*n* = 5) M2 macrophages, cells were lysed and immunoblotted for XBP1s and HPRT. All data is presented as mean ± SEM. Data in panel F are represented by logarithmic scale base 10. All n values represent biological independent samples. Statistical comparisons were performed by Mann-Whitney non-parametric test, ^*^p < 0.05, ^**^p < 0.01, ^***^p < 0.001 **(B–E)**; unpaired independent student's *t*-test, ^*^p < 0.05, ^**^p < 0.01, ^***^p < 0.001 **(F,I)**.

Monocytes are the natural precursors of macrophages, when these are recruited to site of inflammation, and these cells were the most affected by ER stress. Two recent studies have reported differences in M1/M2 macrophage polarization in CF, with contradicting conclusions (12, 13), suggesting further investigations are required to resolve this phenomenon. To do this we used freshly isolated monocytes from CF patients or HC volunteers and differentiate them into either a M1 (CD14+, CD274+, CD86+, HLA-DR+ and Hi TNF) or M2 (CD14+, CD206+, HLA-DR- and Hi IL-10) phenotype as described in Figure 3A. After polarization, we observed a significantly lower amount of M2 macrophages in patients with CF with lower amounts of IL-10 (Figures 3B,C). While the proportion of CF M1 macrophages was unaffected, the amount of IL-6 produced by these proinflammatory cells was significantly increased (Figures 3D,E). As seen in the HBECs, and also in alveolar macrophages (AM) (8), the upregulation of XBP1s is proportional to the exaggerated inflammatory response seen with IL-6 (Figures 1E–G). We found that in CF M1 macrophages *XBP1s* was significantly increased, and correlated with the upregulation of *TNF, IL-6, BiP*, and *ERdj4* (Figure 3F); furthermore these changes were still observed after a second challenge of LPS, with the exception of *ERdj4*, and the downregulation of *PERK* (Figure 3F).To further support the finding that XBP1s is present in CF M1 macrophages, we performed a conventional reverse transcription (RT)-PCR to detect splicing of XBP1. We consistently detected XBP1s in the majority of M1 macrophages from CF patients (Figure 3G). We also detected a significantly higher production of XBP1s at the protein level in CF M1 macrophages, while XBP1s was not detected in M2 macrophages (Figures 3H–J). Taken together, these results support a defect in CF M2 macrophage polarization, demonstrating the existence of ER stress in CF M1 macrophages, with a potentially distinctive role of XBP1s driving this exaggerated inflammatory response.

### Metabolic Profile of Monocytes and M1 Macrophages

**Figure 4:**
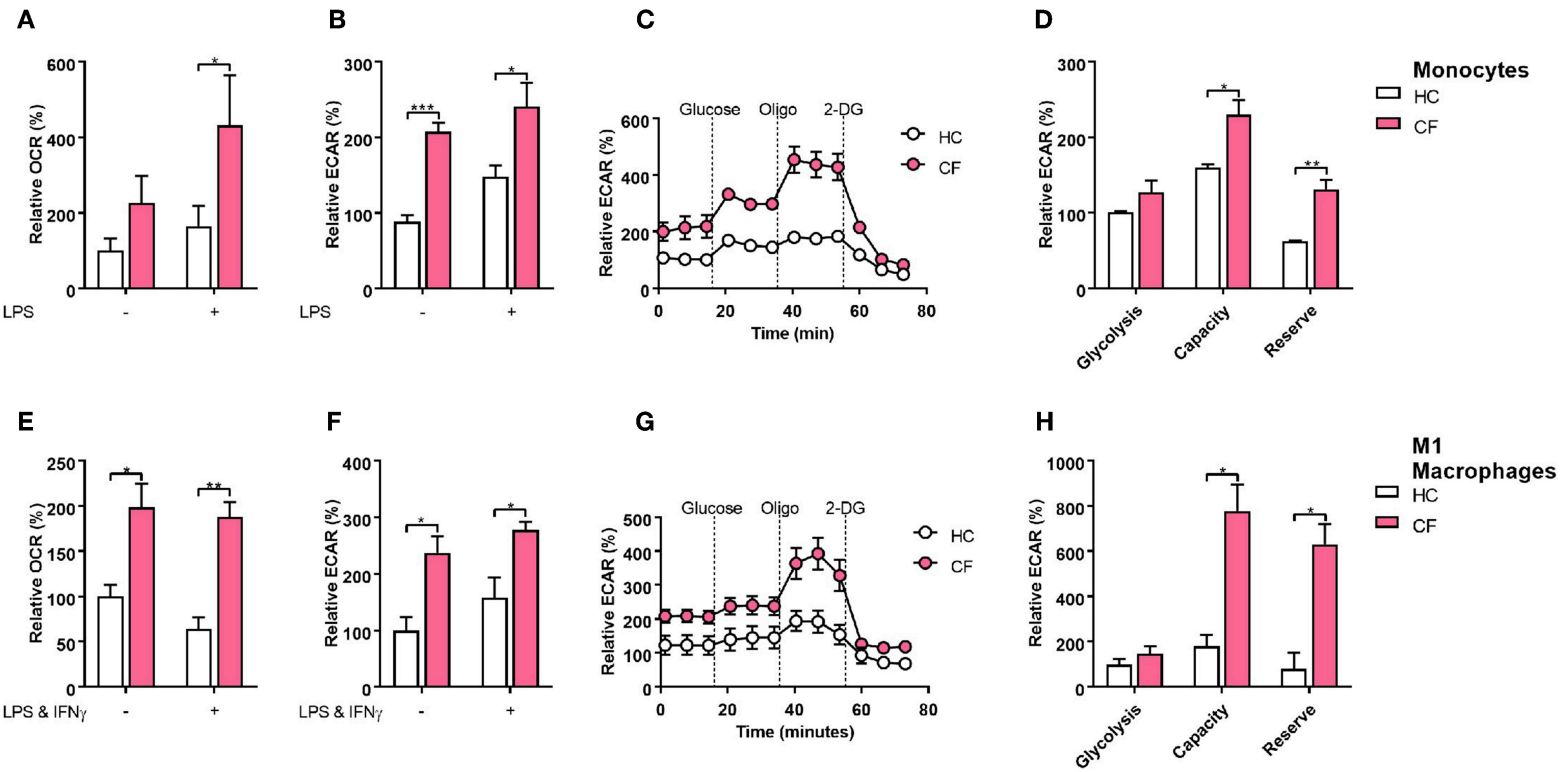
Glycolytic profile of CF monocytes and M1 macrophages. Real time ECAR and OCR of monocytes and M1 macrophages stimulated with glucose (10 mM), oligomycin (1 μM), and 2-Deoxy-D-glucose (2-DG, 50 mM). **(A–D)** Measurement of relative OCR **(A)** or ECAR **(B)** in monocytes from HC volunteers (*n* = 7) and patients with CF (*n* = 7) at basal conditions and stimulated with LPS (10 ng/ml) for 4 h. Real time ECAR levels, glycolysis, glycolytic capacity and reserve **(C,D)**. Representative of 3 HC and 3 CF patients **(C)**. **(E–H)** Measurement of relative OCR **(E)** or ECAR **(F)** in M1 macrophages from HC volunteers (*n* = 7) and patients with CF (*n* = 7) at non-activated conditions and activated with LPS (50 ngml) and IFNγ (100 ng/ml). Real time ECAR, glycolysis, glycolytic capacity and reserve **(G,H)**. Representative of 3 HC and 3 CF patients **(G)**. Glycolysis, glycolytic capacity, and reserve were calculated as described in the methods. The 7 CF patients and 7 HC volunteers in **(A–D)**, are independent from the other panels. All data is presented as mean ± SEM. All n values represent biological independent samples. Statistical comparisons were performed by unpaired student's t-test, ^*^p < 0.05, ^**^p < 0.01, ^***^p < 0.001.

When macrophages are activated toward a M1 proinflammatory phenotype, they increase the level of ATP production by switching to glycolytic metabolism (17). Recent publications have demonstrated that the IRE1α-XBP1 pathway regulates M1/M2 macrophage polarization controlling mitochondrial activity and energy consumption in immune cells (4, 14). It is well accepted that patients with CF present an energy imbalance with an increased energy consumption, which is compensated with a hyper-caloric diet. We speculated that the IRE1α-XBP1 pathway was involved in this increased energy consumption, seen in CF patients, with further repercussions in M1/M2 polarization, metabolism and inflammation. The glycolytic rate and mitochondrial function of immune cells can be calculated in real time by measuring the extracellular acidification rate (ECAR) and the oxygen consumption rate (OCR), accordingly. At basal conditions the glycolytic rate of monocytes, but not mitochondrial function, was significantly increased in patients with CF compared to HC volunteers (Figures 4A,B). As expected, stimulation of monocytes with LPS increased their glycolytic rate and mitochondrial function in both HC and patients with CF, with larger changes in the glycolytic rate and mitochondrial function in CF monocytes (Figures 4A,B). We also observed an increase in the glycolytic capacity and reserve of CF monocytes compared to HC monocytes, with no significant change in glycolysis (Figures 4C,D). Next, we measured the glycolytic rate and mitochondrial function of M1 macrophages, derived from isolated monocytes and cultured as described previously (Figure 3A). We noticed that M1 macrophages from CF patients displayed a higher glycolytic rate and mitochondrial function as compared to the HC M1 macrophages (Figures 4E,F). Furthermore, these altered metabolic changes were detected before and after activation of M1 macrophages (Figures 4E,F). Finally, the glycolytic capacity and reserve of CF M1 macrophages were also elevated as seen in the CF monocytes (Figures 4G,H). Following on from these findings we analyzed the expression of some key metabolic enzymes, hexokinase 2 (HK2), pyruvate dehydrogenase kinase 4 (PDK4), 6-phosphofructo-2-kinase/fructose-2,6-biphosphatase (PFKFB1), estrogen related receptor alpha 1 (ESRRA), peroxisome proliferator activated receptor alpha (PPARA), and uncoupling Protein 3 (UCP3), which are involved in glycolysis and mitochondrial function. To our surprise we detected a significant downregulation of HK2 and PPARA in CF M1 macrophages, compared to the HC, with no differences in PDK4, PFKB1, ESRRA, and UCP3 at the mRNA level (Supplementary Figure 3). These results demonstrate that monocytes and M1 macrophages from patients with CF present an altered metabolic profile with increased ECAR and OCR levels, which are qualitative indicators of mitochondrial function and glycolytic rate, respectively. We also observed a larger glycolytic capacity and reserve in these myeloid cells from patients with CF. While the transcription levels of HK2 and PPARA were unexpectedly downregulated, this might indicate that the high ECAR and OCR levels present in CF M1 macrophages could be due to an increased activity of these enzymes, rather than an increase in protein expression, further work, which is outside the scope of the study, is required to test this hypothesis.

**Figure 5:**
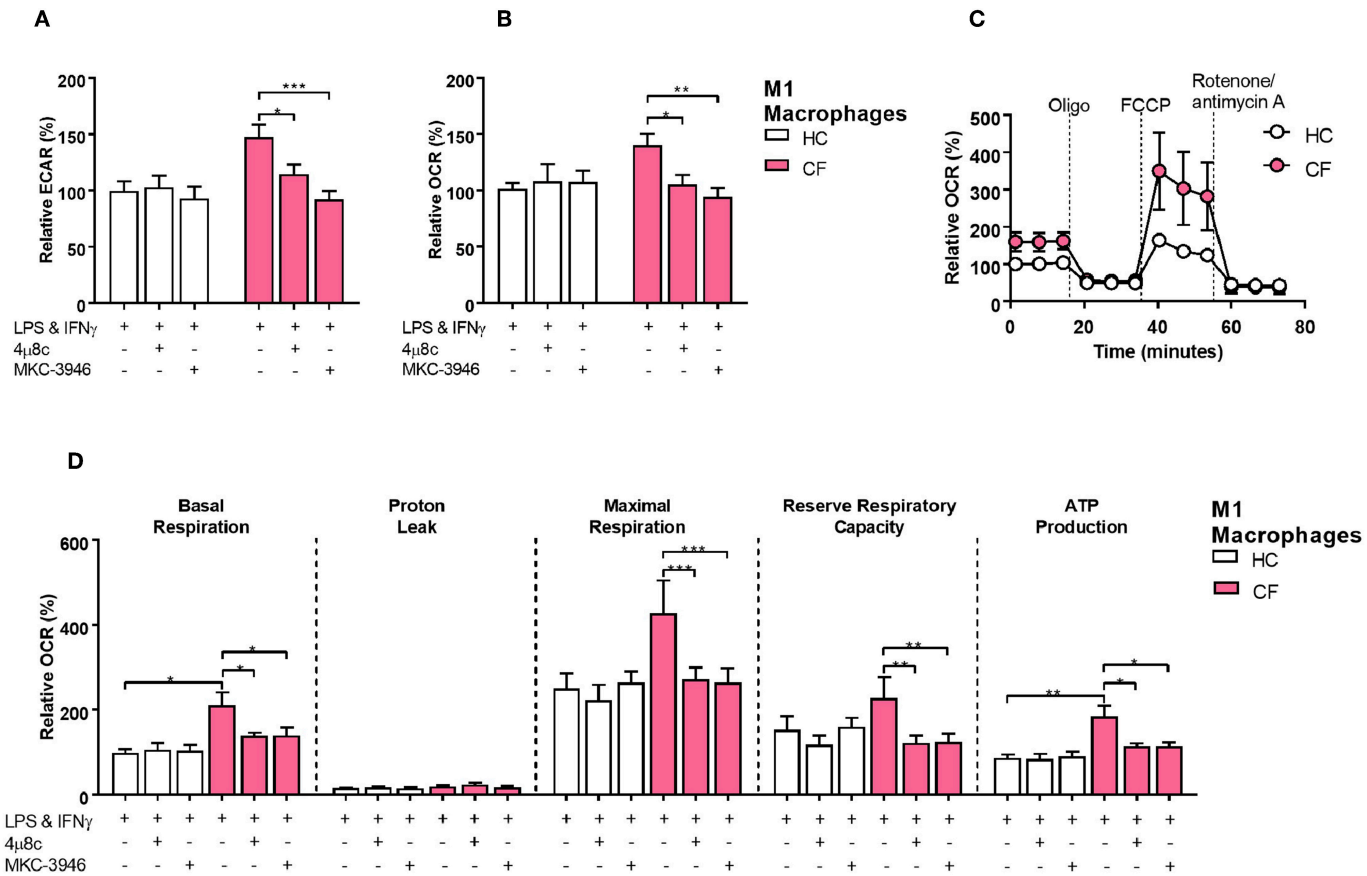
The IRE1α-XBP1 pathway regulates metabolism in CF M1 macrophages. Real time ECAR and OCR of M1 macrophages stimulated with oligomycin (1 μM), FCCP (1 μM), and rotenone/antimycin A (0.5 μM). IRE1α inhibitors 4 μ8c (50 μM) and MKC-3946 (10 μM) were administrated 30 min before M1 macrophages activation. **(A,B)** Measurement of relative ECAR **(A)** or OCR **(B)** in M1 macrophages from HC volunteers (*n* = 9) and patients with CF (*n* = 9). **(C,D)** Real time OCR, basal respiration, proton leak, maximal respiration, reserve capacity, and ATP production. All the values were calculated as described in the methods. Same CF patients and HC volunteers were used in all panels and are the same subjects shown in Figure 6. All data is presented as mean ± SEM. Statistical comparisons were performed by paired or unpaired student's *t*-test, ^*^p < 0.05, ^**^p < 0.01, ^***^p < 0.001.

**Figure 6:**
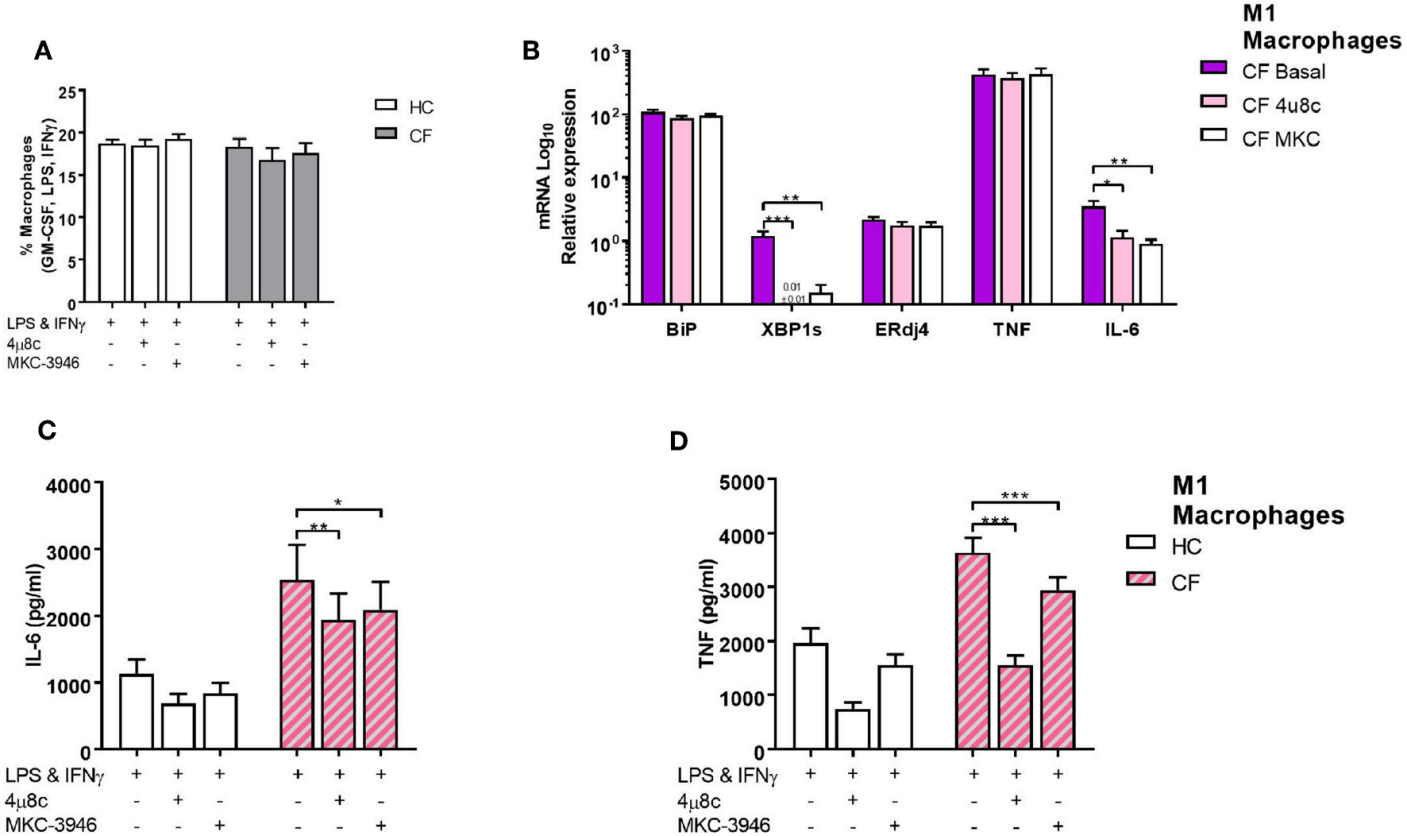
The IRE1α-XBP1 pathway regulates inflammatory cytokine secretion in CF M1 macrophages. IRE1 inhibitors 4 μ8c (50 μM) and MKC-3946 (10 μM) were administrated 30 min before M1 macrophages activation. **(A)** Polarized M1 macrophages from HC volunteers (*n* = 9) and patients with CF (*n* = 9) are presented as percentage M1 of total macrophages measured by flow cytometry. **(B)** mRNA relative expression of ER stress and UPR markers *BiP, XBP1s, ERdj4,TNF*, and *IL-6* in M1 macrophages from HC volunteers (*n* = 6) and patients with CF (*n* = 6). **(C,D)** IL-6 and TNF cytokine levels from M1 macrophages measured by ELISA, HC volunteers (*n* = 6) and patients with CF (*n* = 6). The same CF patients and HC volunteers were used in all panels. All data is presented as mean ± SEM. Data in panel B are represented by logarithmic scale base 10. Statistical comparisons were performed by paired student's *t*-test, ^*^p < 0.05, ^**^p < 0.01, ^***^p < 0.001.

We next investigated whether activation of the IRE1α-XBP1 pathway, which was responsible for the high levels of XBP1s, had an influence in the increased metabolic state of CF M1 macrophages. As seen before the IRE1α-XBP1 pathway was significantly increased in CF M1 macrophages, with increased levels of *TNF* and *IL-6* (Figure 3F). By inhibiting the IRE1α-XBP1 pathway, using specific IRE1α inhibitors, 4 μ8c and MKC-3946, before activation of M1 macrophages, we observed a reduction in the glycolytic rate of 32.7 and 55.0%, respectively (Figure 5A). Similarly, the increased mitochondrial rate of CF M1 macrophages was reversed by the two inhibitors, reducing the OCR of CF M1 macrophages by 34.5%, with 4 μ8c, and 45.5%, with MKC-3946, comparable to the levels of HC M1 macrophages (Figure 5B). We then measure different metabolic parameters using modulators of mitochondrial respiration targeting key components of the electron transport chain to measure cellular basal respiration, proton leak, maximal respiration, reserve respiratory capacity and ATP production. We found that CF M1 macrophages had a significant higher basal respiration and ATP production compared to HC M1 macrophages (Figures 5C,D). Remarkably, the two IRE1α inhibitors significantly reduced basal respiration, maximal respiration, reserve respiratory capacity, and ATP production in CF M1 macrophages (Figure 5D). Surprisingly, we did not observe any differences in the polarization ratio of M1 macrophages when the two IRE1α inhibitors were administrated (Figure 6A). To confirm that inhibition of the IRE1α-XBP1 pathway reduced the inflammatory phenotype of M1 macrophages, we measured IL-6 and TNF at the transcriptional and protein level. As expected, the two inhibitors showed their potent suppressive effects on *XBP1s* (Figure 6B); however, inhibition of the IRE1α-XBP1 pathway only reduced the transcription levels of IL-6, but not TNF in CF M1 macrophages (Figure 6B). Moreover, no differences were detected in the levels of BiP or ERdj4 (Figure 6B). In accordance with the *IL-6* transcript levels, the two inhibitors significantly reduced the amount of IL-6 produced by CF M1 macrophages (Figure 6C). Even though *TNF* was not reduced at the mRNA level, both inhibitors showed a strong TNF suppressive effect at the cytokine level, with the 4 μ8c inhibitor showing a more potent effect (Figure 6D). Together, these results demonstrate that the IRE1α-XBP1 pathway is mainly responsible for the hyper-metabolic state of CF M1 macrophages, which caused an increased inflammatory response.

## Discussion

**Figure 7:**
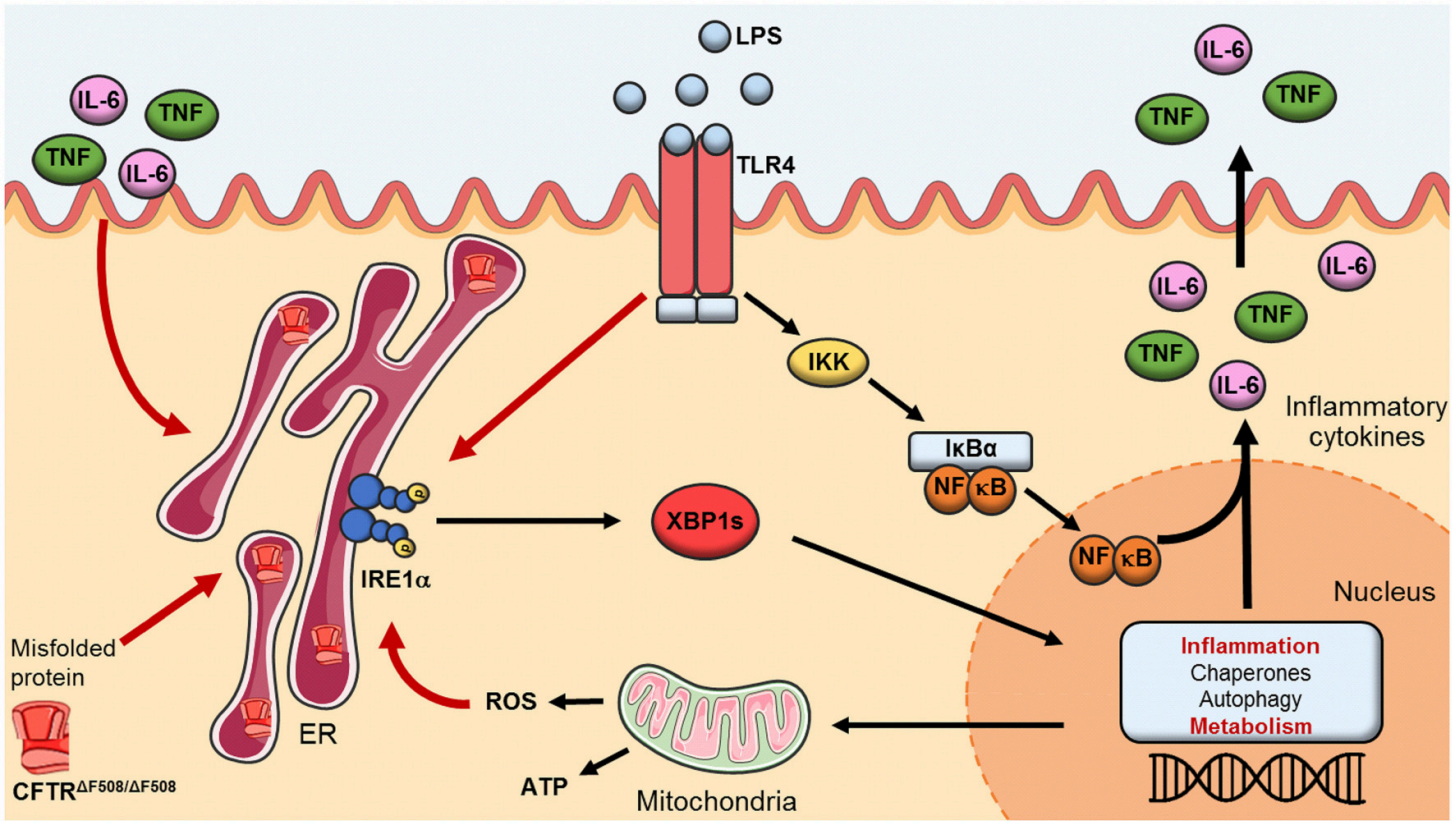
The IRE1α-XBP1 pathway of the UPR in CF. Activation of the IRE1α-XBP1 pathway in macrophages leads to splicing of XBP1 (XBP1s) which activates transcription of genes involved in inflammation, protein folding, autophagy and metabolism. In CF macrophages malfunctioning and accumulation of the misfolded CFTR induces a perpetual ER stress, leading to heightened levels in metabolic functions and chronic low-grade inflammation. TLR4 activation, through LPS, leads to a hyper-inflammatory/metabolic response through XBP1s exacerbating the inflammatory response.

Macrophages are versatile cell types capable of controlling the inflammatory response, as may be required by the prevailing physiological environment. M1 macrophages promote an inflammatory response by secreting large amounts of proinflammatory cytokines when polarized, while M2 macrophages are responsible for the resolution of inflammation with large secretion of anti-inflammatory cytokines. CF has been described as an autoinflammatory condition, due to the lack of involvement of the adaptive immune system and an overwhelming and chronic inflammatory response mainly driven by the innate immune system (18). Autoinflammatory conditions have been described as a self-directed inflammation influenced by the local tissue environment leading to activation of innate immune cells causing tissue damage and cellular death (19). We demonstrated that patients with CF present unique UPR activation, which is differentially upregulated in HBECs, neutrophils, monocytes and M1 macrophages, while being completely absent in adaptive immune cells. These findings provide evidence to suggest that CF could be considered as an autoinflammatory condition driven by the elevated ER stress seen in innate immune cells with further repercussions in inflammation. We have demonstrated that the IRE1α-XBP1 pathway induces a chronic degree of low-grade inflammation in CF M1 macrophages, perhaps due to the accumulation of misfolded CFTR in the ER, and this inflammatory response can be exacerbated by further stimulation with bacterial components, such as LPS (Figure 7). Similar findings were found in autoinflammatory patients with Tumor Necrosis Factor Receptor Associated Periodic Syndrome (TRAPS), characterized by intracellular accumulation of misfolded proteins, with enhanced IRE1α activity and inflammation (20, 21). Furthermore, spontaneous lung and pancreatic inflammation have been reported in several animal models of CF, and in some models even in the absence of infection (22–24). These observations support the idea that CF drives an autoinflammatory phenotype, perhaps due to an increase in ER stress, with a consequential formation of fibrotic tissue. While the only differences we observed in CF PBMCs under basal conditions were *IRE1*α, *GADD34*, and *IL-6*, contrary to the differences found by Blohmke et al. (15), we observed changes in a greater number of transcripts following stimulation with the bacterial component, LPS, suggesting that bacterial infections are the main environmental factors triggering ER stress and inflammation in CF. The unexpected downregulation of *IL-6*, in CF PMBCs, could be due to resolution of the inflammatory response, as it was significantly higher after stimulation with LPS (Figure 2A). Furthermore, there is a unique ER stress signature in different subsets of immune cells, with consistent upregulation of *IRE1*α. Despite the fact that neutrophils possess a rudimentary ER, due to their biological functions and short lifespan, we did observe a significant upregulation of *BiP, IRE1*α*, ATF4*, and *CHOP* in these phagocytic cells. These findings suggest an important role of these molecules in neutrophil regulation, perhaps affecting their phagocytic or inflammatory activity. Monocytes were the cells most affected by ER stress, with consistent upregulation of *IRE1*α, *ERdj4, PERK, ATF4, GADD34*, and *TNF* but without appreciable differences in *XBP1s* expression. Interestingly, the significant upregulation of *XBP1s*, detected in CF PBMCs after LPS stimulation, was not observed in lymphocytes, neutrophils or monocytes, suggesting a possible mechanistic interaction of immune cells to influence the regulation of UPR genes. As monocytes are being differentiated toward macrophages, their ER is expanded, and their cellular size becomes enlarged, thereby providing these cells with a greater capacity to promote inflammation and phagocytosis. As previously reported (12), we observed a reduced of polarization of M2 macrophages in patients with CF, which could be due to increased levels of ER stress in these anti-inflammatory cells. We did not detect any polarization abnormality in M1 macrophages; however, CF M1 macrophages displayed an increased activity of the IRE1α-XBP1 pathway, with increased *BiP, XBP1s, ERdj4, TNF*, and *IL-6*. Macrophages are phagocytic cells directly involved in the clearance of pathogens. It has been shown that macrophages with CF mutations present altered phagocytic functions and bacterial killing (25–27). While a direct connection between IRE1α activation and phagocytosis has not been investigated, it is worth mentioning that XBP1s is directly involved in the regulation of autophagy, by transcriptional activation of BECLIN-1 (28). Furthermore, when XBP1s is upregulated during bacterial challenge it will induce the upregulation of components involved in autophagy; hence the lysosomes will be utilized rapidly by the three main processes involved in degradation of different components, namely endocytosis, phagocytosis and autophagy, leading to deficient bacterial killing and autophagy. It will be of great interest to investigate whether the IRE1α-XBP1 pathway is involved in phagocytosis not only in CF, but also in other disorders.

In line with previous investigations showing that the IRE1α-XBP1 pathway is involved in maintaining the metabolic fitness of immune cells (4), we found that this pathway regulates metabolism in CF M1 macrophages. In our studies, we observed an increased ECAR in CF monocytes, under basal conditions, while both ECAR and OCR were largely increased in CF monocytes after LPS stimulation, compared to HC volunteers. This indicates that both the mitochondrial and glycolytic function of CF monocytes were significantly increased when challenged to bacterial components. Similarly, CF M1 macrophages displayed the same hyper-metabolic state before and after polarization, suggesting that these abnormalities are kept after macrophage polarization and in the absence of bacterial infections. Furthermore, monocytes and M1 macrophages presented an increased glycolytic capacity and reserve, which could be due to the increased ER stress. These findings support the idea that cells with CF mutations are adapted to chronic ER stress, exacerbated by bacterial infections, improving their ability to generate ATP. The increased levels in the glycolytic rate and mitochondrial activity of CF M1 macrophages were reversed by inhibition of the IRE1α-XBP1 pathway. This indicates that this pathway is metabolically reprograming CF M1 macrophages toward a hyper-metabolic state directly associated with the heightened inflammatory response. The two inhibitors, 4μ8c and MKC-3946, showed a potent inhibitory effect in the proinflammatory cytokines IL-6 and TNF. We did not detect any changes in the polarization of M1 macrophages when the inhibitors were administrated, perhaps due to the chronic ER stress present in CF cells or, possibly, due to the surface markers we used to characterize the macrophages. It is important to mention that suppression of the RNase domain of IRE1α, with the 4 μ8c and MKC-3946 inhibitors, not only decreases XBP1s, but also several other important transcripts involved in cellular homeostasis (29). Inhibition of the RNase domain of IRE1α also interferes with the regulated IRE1-dependent decay (RIDD) pathway (29); it has been shown that RIDD regulates around 120 transcripts in mammalian cells, with profound effects on protein production and cellular fitness (30). For example, dermal fibroblasts from patients with TRAPS fail to upregulate miR-146a and miR-155, due to an increased activity in IRE1, which can be reversed by inhibition of the RNase domain of IRE1α, with 4 μ8c, thereby restoring the levels of miR-146a and miR-155 (21). Another study showed that sustained activation of RIDD influenced the cleavage of miR-17, miR-34a, miR-96, and miR-125b, by repressing translation of Caspase-2 mRNA, which controls the induction of apoptosis under chronic ER stress (31). It is clear that RIDD is an important transcriptional regulator involved in cellular homeostasis and fitness. It will be of great interest to investigate whether the transcripts, targeted by RIDD during ER stress in CF M1 macrophages, are linked to the abnormal metabolic and inflammatory levels shown here.

In summary, activation of the IRE1α-XBP1 pathway in CF M1 macrophages leads to a hyper-metabolic state, with high levels of ECAR and OCR, associated with a heightened inflammatory repose. The exaggerated inflammatory response seen in patients with CF has been well established before, but it has never been linked to an increased metabolic profile of the classically activated macrophages. M1 macrophages from CF patients undergo metabolic reprogramming, through the IRE1α-XBP1 pathway, leading to high mitochondrial and glycolytic activity, with associated increased levels of TNF and IL-6. These findings help to explain why patients with CF suffer from chronic and unresolved inflammation, not only present in the lungs but also in joints, pancreas and gut (32–36). Malfunctioning of the CFTR, along with accumulation of the misfolded channel, in the case of ΔF508 homozygous patients, induces a perpetual chronic low-grade inflammation in CF macrophages, predisposing these cells to a hyper-inflammatory and metabolic response, driven by XBP1s, which is exacerbated by the activation of TLR4 (Figure 7) and may be unresolved due to the lack of M2 macrophage polarization. It is worthwhile mentioning that all CF patients in our study were clinically stable, with a mean BMI of 23.1 and FEV1 of 45.9% (Table 1), indicating that our findings are not due to the presence of clinical complications, but rather to the CF mutations present in macrophages. Our findings are in agreement with several investigations in the field of CF and metabolism, which showed that the increased glycolytic rates and mitochondrial activity were responsible for the abnormal inflammatory responses. This connection is important in the context of inflammation and metabolism, not only in CF, but in other disorders also, showing that modulation of ER stress, through inhibition of the IRE1α-XBP1 pathway, may help to recover the metabolic fitness of immune cells in this debilitating condition.

## Data Availability

All datasets generated for this study are included in the manuscript and/or the Supplementary Files.

## Ethics Statement

All work involving human samples from patients with CF or HC volunteers was approved by the Health Research Authority, Research Ethics Committees reference 17/YH/0084.

## Author Contributions

SL-R, TS, FM, SS, DP, and MM: methodology. SL-R, TS, and CW: validation, formal analysis, and data curation. SL-R, TS, CW, JH, HJ-G, SS, DP, and MM: investigation and writing–review and editing. SL-R, TS, SS, DP, and MM: resources and funding acquisition. SL-R: writing–original draft. SL-R and TS: visualization. SS, DP, and MM: supervision.

### Conflict of Interest Statement

The authors declare that the research was conducted in the absence of any commercial or financial relationships that could be construed as a potential conflict of interest.
