# Supplementary material for: Metabolic Reprograming of Cystic Fibrosis Macrophages via the IRE1α Arm of the Unfolded Protein Response Results in Exacerbated Inflammation
Source: Front Immunol. 2019 Aug 2;10:1789. doi: 10.3389/fimmu.2019.01789 (PMC6687873; doi:10.3389/fimmu.2019.01789)
Supplement: Supplementary file 1 [file Data_Sheet_1.PDF]

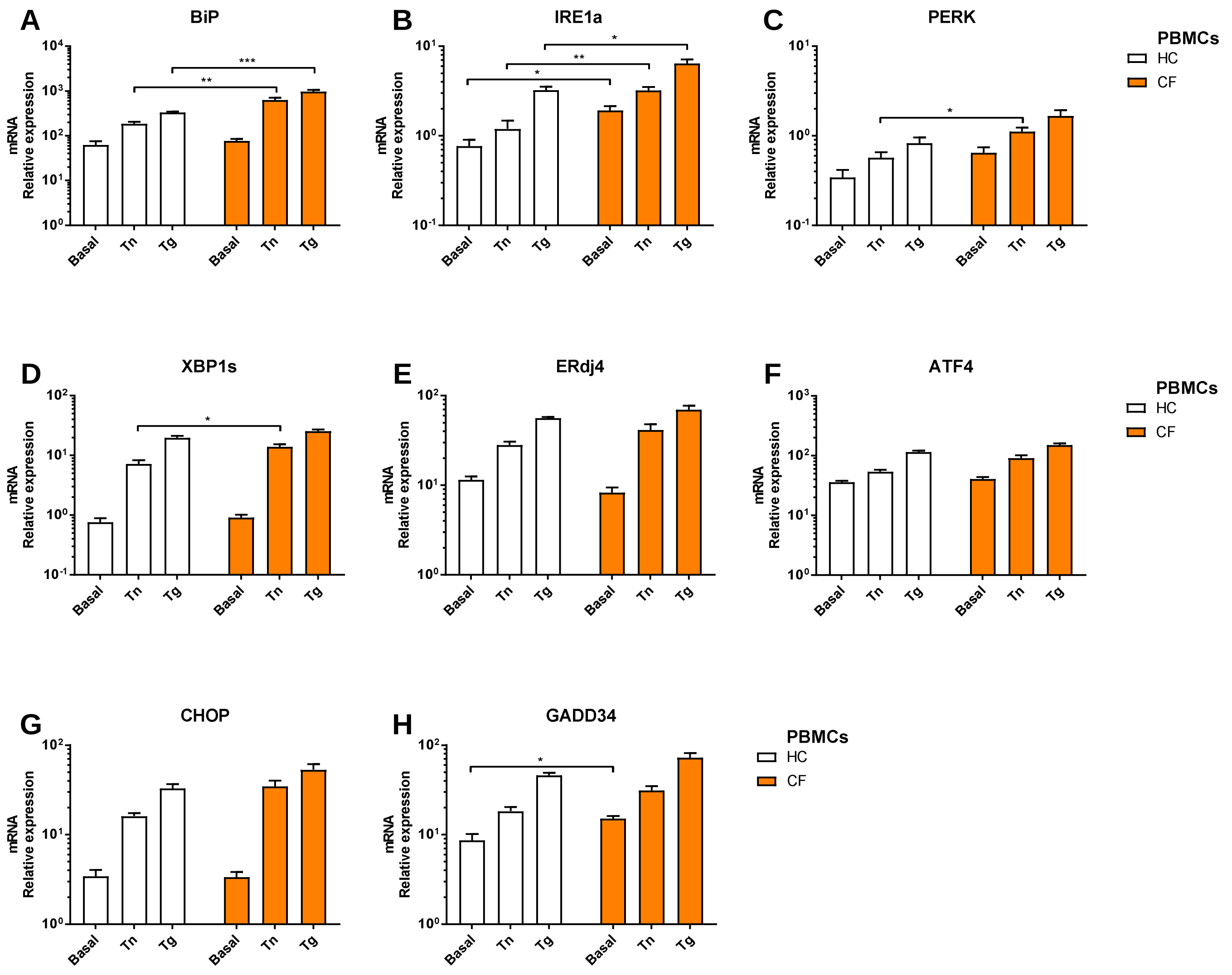

Supplementary Figure 1. ER stress in PBMC stimulated with Tn and Tg. mRNA relative expression of ER stress and UPR markers *BiP, IRE1α, PERK, XBP1s, ERdj4, ATF4, CHOP* and *GADD34* in primary PBMCs from HC individuals (n=8) and patients with CF (n=14) at basal conditions, stimulated with Tunicamycin (Tn) (5 µg/ml), or Thapsigargin (Tg) (300nM) for 4 hours. (A-H). All the subjects presented in here are the same as those presented in Figure 1A. All data is presented as mean ± SEM and mRNA data represented by logarithmic scale base 10. Statistical comparisons were performed by unpaired independent student's t-test. *$p < 0.05$, **$p < 0.01$, and ***$p < 0.001$.

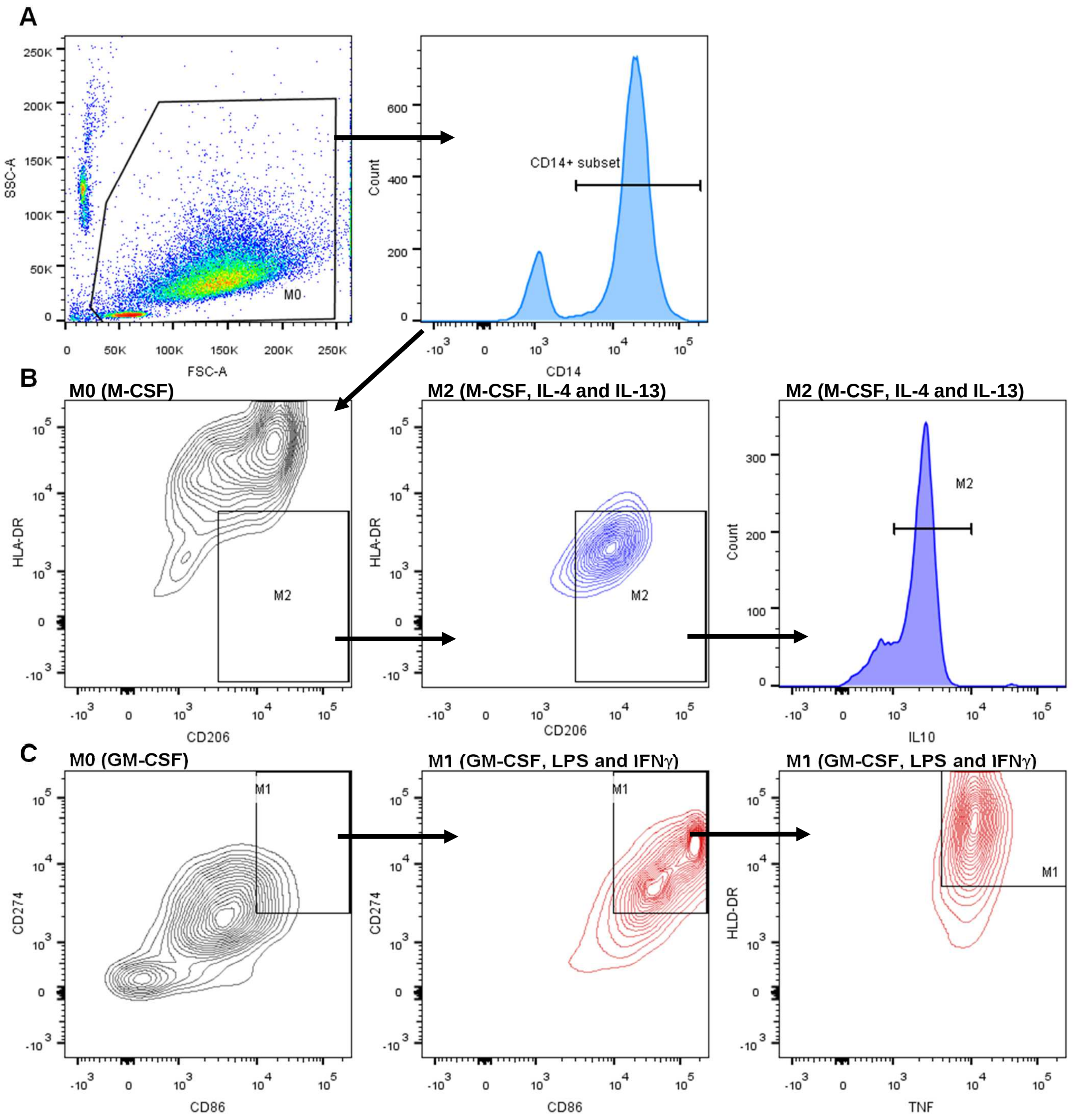

Supplementary Figure 2. M1/M2 Macrophage Gating Strategy. Representative gating strategy for M1/M2 macrophage characterization. Gating started on positive selection of all the potential M1/M2 macrophages done by SSC/FSC. Then CD14+ macrophages were selected (A). CD14+ macrophages were considered M2 macrophages only when they were HLA-DR-,CD206+, and IL-10+ (B). CD14+ macrophages were considered M1 macrophages only when they were CD274+, CD86+, HLA-DR+ and TNF+. All antibodies used are listed in detail in the resource table.

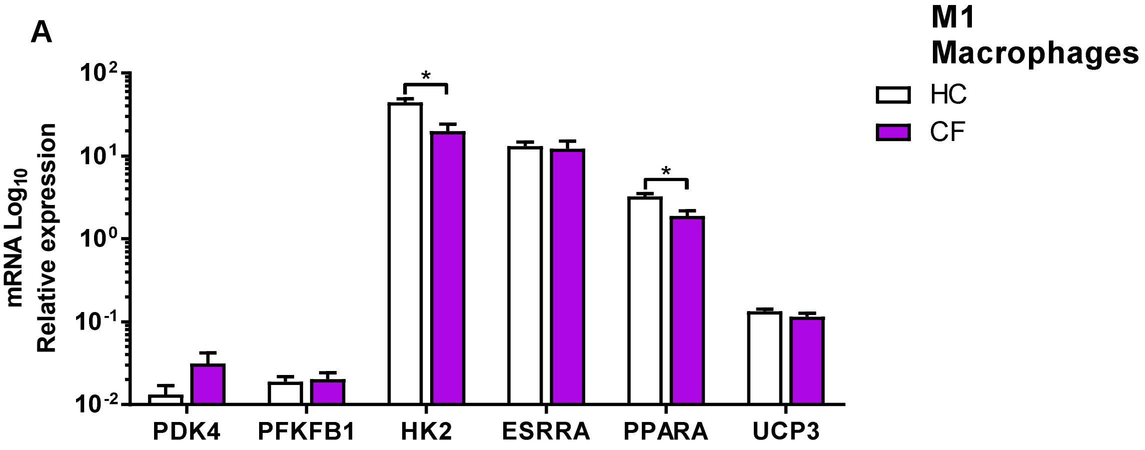

Supplementary Figure 3. Key metabolic enzymes in M1 macrophages. mRNA relative expression of PDK4, PFKB1, HK2, ESRRA, PPARA, and UCP3 in M1 macrophages from HC volunteers (n=5) and patients with CF (n=5). All data is presented as mean ± SEM and mRNA data represented by logarithmic scale base 10. Statistical comparisons were performed by unpaired independent student's t-test. $*p < 0.05$, $**p < 0.01$, and $***p < 0.001$.

Gene Target	Forward Sequence (5'->3')	Reverse Sequence (5'->3')
BiP	GAACGTCTGATTGGCGATGC	TCAACCACCTTGAACGGCAA
IRE1α	TAGTCAGTTCTGCGTCCGCT	TTCCAAAAATCCCGAGGCCG
PERK	GCGCGGAAAGTTTGCTCAAT	GAGCTCCCAAGAAGGCAAGG
ATF6	ATGAAGTTGTGTCAGAGAACC	CTCTTTAGCAGAAAATCCTAG
XBP1s	CTGAGTCCGCAGCAGGTG	AGTTGTCCAGAATGCCCAACA
XBP1u	TCCGCAGCACTCAGACTACG	AGTTGTCCAGAATGCCCAACA
ERdj4	GTCGGAGGGTGCAGGATATTAG	GCGCTCTGATGCCGATTTTG
CHOP	GGAACCTGAGGAGAGAGTGTT	GTCCCGAAGGAGAAAGGCAA
GADD34	CTGGCTGGTGGAAGCAGTAA	TATGGGGGATTGCCAGAGGA
ATF4	GCCAAGGGGGAAGCGATTTA	CTACGCTTTCCCGATCCCAG
IL-6	CCAGCTATGAACTCCTTCTC	GCTTGTTCCTCACATCTCTC
TNF	CACCACTTCGAAACCTGGGA	TGTAGGCCCCAGTGAGTTCT
HPRT	GGAAAGAATGTCTTGATTGTGGAAG	GGATTATACTGCCTGACCAAGGAA
PDK4	Assay ID: Hs01037712_m1	
PFKB1	Assay ID: Hs00997227_m1	
HK2	Assay ID: Hs00606086_m1	
ESRRA	Assay ID: Hs00607062_gH	
PPARA	Assay ID: Hs00947536_m1	
UCP3	Assay ID: Hs01106052_m1	
HPRT	Assay ID: Hs02800695_m1	

Supplementary Table 1. Primer Sequences.
